# Automation, Climate Change, and the Future of Farm Work: Cross-Disciplinary Lessons for Studying Dynamic Changes in Agricultural Health and Safety

**DOI:** 10.3390/ijerph20064778

**Published:** 2023-03-08

**Authors:** Matt Comi, Florence Becot, Casper Bendixsen

**Affiliations:** National Farm Medicine Center, Marshfield Clinic Research Institute, Marshfield, WI 54449, USA

**Keywords:** automation, agriculture, climate change, farm work, health, safety, futures

## Abstract

In this review, we first assess the state of agricultural health and safety research as it pertains to the dynamic challenges facing automating agriculture on a warming planet. Then, we turn to social science fields such as rural sociology, science and technology studies, and environmental studies to leverage relevant insights on the introduction of new technologies, environmental risks, and associated workplace hazards. Increased rates of automation in agriculture alongside new risks associated with climate change create the need for anticipatory governance and adaptive research to study novel mechanisms of worker health and safety. The use of the PRISMA framework led to the 137 articles for our review. We identify three themes in the literature on agricultural health and safety: (1) adoption outcomes, (2) discrete cases of health risks, and (3) an emphasis on care and wellbeing in literature on dairy automation Our review led to the identification of research gaps, noting that current research (a) tends to examine these forces separately, instead of together, (b) has not made robust examination of these forces as socially embedded, and (c) has hesitated to examine the broad, transferable themes for how these forces work across industries. In response to these gaps, we suggest that attention to outside disciplines may provide agricultural health and safety research with a toolset to examine needed inquiry into the multiplicity of experiences of rural stakeholders, the industry specific problems arising from automation and climate change, and the socially embedded aspects of agricultural work in the future.

## 1. Introduction

Climate change and automation pose unique challenges to agricultural health and safety. This is compounded by a reliance on fossil fuels, making agriculture a driver and a victim of global economic and environmental pressures [1,2,3]. A key question is then, how can research and policy account for the dynamic pressures of these macro-level challenges in shaping agricultural populations’ exposure to risks considering that the impacts of climate change and rapid automation vary locally across geographies and commodities? While the field of agricultural health and safety has studied both the role of weather and the role of automation in shaping agricultural populations’ health and safety outcomes [4,5,6,7], climate change and rapid automation are dynamic, not static, forces—bringing on novel challenges, such as higher heat, less predictable local weather patterns, new plant, chemical, and machine technologies; all of which augment the nature, safety, and risks associated with farm work. Increasing global surface temperature has dramatically shifted local environments and produced a number of agricultural challenges from crop adaptability to systems resilience and to owner/operator and worker health and safety [8,9,10,11,12]. In parallel, increasing automation has been a hallmark of the industrialization and digitalization of agriculture, promising to lower the negative health, safety, and economic externalities of labor in agriculture [11,13]. While these trends impact future trajectories for agriculture, the industry remains highly dangerous [14] with workers, farm operators, and their families facing exposure to hazardous chemicals, equipment, and environmental conditions [15,16,17].

Understanding these dynamic and complex challenges will require observing relationships between environment, technique, and health risk exposures. The agricultural health and safety field is particularly suited for such observation given its disciplinary grounding in engineering, health sciences, and public health. In addition, a deeper understanding will require analysis of social structures, cultural factors, and attitudinal shifts that relate to and inform technological and environmental change. Over the years, social scientists have enhanced the agricultural health and safety field by studying the impact of mechanization and industrialization on health, risk behavior, the social determinants of rural health, particularly rural health inequalities [18,19,20,21]. Their research addressing environmental change, agri-food tech, and science and technology more generally holds still more relevance for understanding the challenges brought on by climate change and rapid automation. In these bodies of work, social scientists have elucidated the intersection of material and social worlds along with the politics, ideologies, and outcomes of dynamic pressures related to innovation and climate change [18,19,20,21].

As a baseline, it is essential for researchers to have a clearer understanding of the existing agricultural health and safety research that examines climate change and automation—to achieve this, we have conducted a scoping literature review designed to explore the contours of the field as currently arranged. We consider both what this scholarship has covered and what it has not covered using a Preferred Reporting Items for Systemic Reviews and Meta-Analysis (PRISMA) framework to map out the work and identify gaps where additional research trajectories may be beneficial. We then summarize key themes using concepts from Environmental Social Science, Science and Technology Studies, and rural social science, while as we will discuss, these social science bodies of literature touch on topics not currently covered by the agricultural health and safety literature they expand the theoretical boundaries of agricultural health and safety to better consider the social structural obstacles and opportunities for safer and healthier farm work in the future. Thus, in the third and last section of our article, we suggest novel synergy between the agricultural health and safety literature and the social science bodies of literature—highlighting how theories explaining the co-constitutions of society, environment, and technologies reframe the impacts of climate and technology change in agricultural health and safety. The resulting paper contributes to disciplinary knowledge by scoping the existent research in the area of agricultural health and safety and provides avenues for productive disciplinary cross-pollination in future research design.

## 2. Literature Review Approach

**Figure 1:**
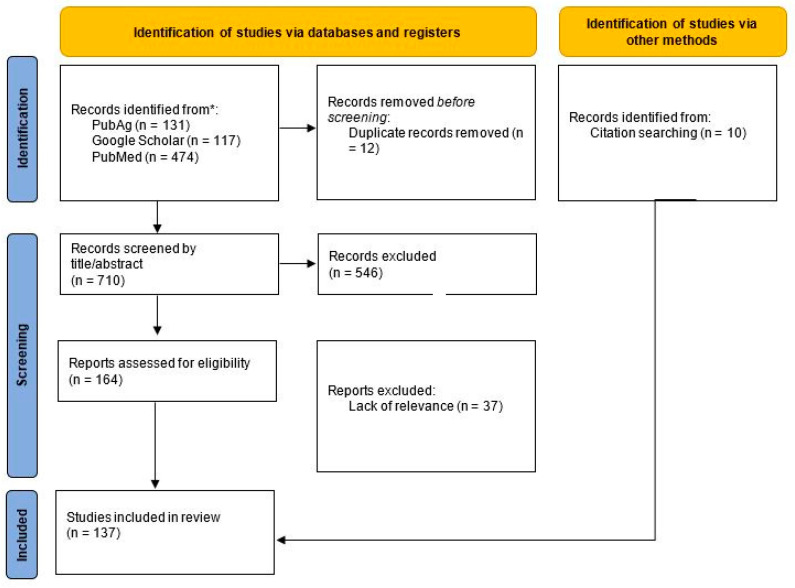
PRISMA Framework used for review. * Records retrieved from databases using Boolean search term: (agriculture or farming) and (automation or technology) and (“climate change” or “environmental change”).

We used the PRISMA framework (see Figure 1) as the basis for this literature review to systematically identify and link relevant agricultural health and safety research with agriculture, technology, and climate change [22]. As we sought links between these topics, our search terms required that a term from each group was present in the search, our Boolean phrase reads (agriculture or farming) and (automation or technology) and (“climate change” or “environmental change”). Searches were performed in PubAg, PubMed, and Google scholar databases and limited to articles published in English over the last ten years (i.e., 2012), two years prior to the 2014 Intergovernmental Panel of Climate Change (IPCC) report calling attention to the important links between land use (e.g., farming and forestry) and climate change. While drawing a specific before and after is somewhat arbitrary, we suggest that this represents a threshold where climate change and land use entered a new degree of attention and a new phase of scholarship.

We identified a total of 710 articles. We conducted an initial screen by assessing the health/safety relevance of titles and abstracts, leaving 164 remaining articles. In a second screen, we reviewed full articles and removed those that did not have sufficient relevance to the topic of health and safety in agricultural work leading to 127 remaining articles. Finally, additional articles were introduced based on citation searches, particularly through existing literature reviews. This process led to the addition of 10 articles. In total, our review results constitute 137 articles. We synthesized key terms emergent in the literature to develop three thematic foci: 1. outcomes related to the adoption of environmental adaptation strategies; 2. discrete causes of farm injuries under climate and technological change; and 3. society, care, and wellbeing in the future of automated farm work. These themes are each explicated below. The production of these themes developed from notes we took on the goals of each study, their content area and geographic scope, and their population focus.

## 3. Technology and Climate Change in the Literature as It Pertains to Agricultural Safety and Health

Notably, our synthesis of these subtopics found health and safety research developing three silos of thematic material. Papers aligning with these themes tend not to cite/interact with the others in ways that substantially inform their key findings. Thus, while these themes should not be read as the total encapsulation of the research field but instead a generalization about the contours of the literature as it stands, it will become clear in later parts of this review how these themes can better illuminate one another’s findings.

### 3.1. Theme 1: Outcomes Related to the Adoption of Environmental Adaptation Strategies

One core consideration of the literature regards how automation and climate-smart agriculture are adapting in response to climate change [23,24,25,26,27,28,29,30,31,32,33,34,35,36,37,38,39,40]. From this literature, a portion focuses on the holistic wellbeing, with an emphasis on economic factors, of farm owner/operators [25,29,33]. Meanwhile, another portion examines the impacts of environmental change on worker health, most notably as that population’s health is impacted by dynamic changes in temperature and high heat exposure [38,39]. Our PRISMA search revealed only a small number of publications focused primarily on climate change and agriculture that also discussed the significant impacts of climate change on the lived experience of farm workers [41,42,43,44,45]. This small number belies the larger body of work that does study farmworkers and implicitly addresses their experiences of climate change in many cases [7,16,44,46,47,48,49,50,51]. However, as described in the Conclusion, it remains clear that the body of literature examining impacts of environmental change and automation on agriculture could further consider those whose lives and wellbeing are most directly affected by these changes. The danger to worker wellbeing compounds when considering that these same populations tend to have the least agency to make their own decisions or negotiations around the adoption, use, and interaction with forces of automation and environment [52]. Of the articles we identified, wellbeing received less attention than health, and from that, only research on automated milking systems examined their impact on farmer wellbeing [26,27,53]. Research on worker wellbeing in relation to automation was not clearly available.

### 3.2. Theme 2: Discrete Causes of Farm Injuries under Climate Change and Technological Change

The second theme regards discrete causes of farm injuries under climate and technological change (e.g., rates of heat related illness). The majority of this work examined outcomes related to heat and repetitive tasks, with an emphasis on extreme heat; a subsidiary group of scholarship examined automated and AI-driven surveillance of these same factors [23,29,34,37,38,39,41,42,43,44,45,53,54,55,56,57,58,59,60,61,62,63,64,65,66,67,68,69,70,71,72,73,74,75,76,77,78,79]. The literature focuses primarily upon the discrete forces shaping negative health outcomes (e.g., the effect repetitive tasks may have on back health, or the impact higher heat may have on heat related illnesses). While literature exists considering the social determinants of health and inequalities farm workers face while exposed to these forces [49,80,81], few draw correlations between social, environmental and technological forces at play on the farm. Notable exceptions hail from the field of anthropology, including Holmes’ [49] study on the naturalization of social suffering among farmworkers, particularly a group of Oaxacan workers with whom he conducted an ethnographic study. His work, alongside that of Arcury and others [46,47,48], highlights the value of social science approaches to the agricultural health and safety field. From this approach, inquiry into the social and political forces in farm work’s material and embodied reality centers and illuminates the changing dynamics in worker health and wellbeing in the context of automation for labor intensive agriculture and climate change’s new health risks.

### 3.3. Theme 3: Automation, Care, and Wellbeing on the Farm

The third theme groups dairy farmers and farmworkers and the impact of automation in their industry with considerations of society, care, and wellbeing in the future of the automated farm. While some overlap exists with aspects of Theme 1, this body of literature highlights inputs (care, attention) and societal-level considerations (e.g., holistic wellbeing, work quality) in the context of climate change and automation. This research also primarily focuses in US and European contexts and considers holistic wellbeing, the physical impacts of farm labor, and the relationship between farmers and farm workers on dairy operations in the changing landscape of agriculture in the last ten years [5,6,53,65,82,83]. Notably, this research has, at times, adopted approaches from other disciplines described below—as is the case of Arcury and Holmes’ work with workers in labor-intensive plant agriculture [46,47,49,81,84].

In both the cases of Arcury and Homes’ respective work, inequalities along class, citizenship, race, and ethnic lines everts into health outcomes on the farm. In the case of dairy research, where behavioral factors and attitudinal concerns about farmers impact wellbeing outcomes for those adopting automations, we see that the material world of farm safety is also socially embedded, and these societal consideration are themselves implicated in specific environments and technological practices. The interplay of these spheres, supposedly discrete in their professional specialties, reveals that multivariate health and safety concerns arise from climate change and automation. However, such interplay also reveals the potential for insights relevant not only to specific industries and cases but also to social science and health research more broadly as principals of behavior, attitude, and social norms play out in cognate areas of health and safety research.

### 3.4. Gaps in the Current Research

We now turn to the gaps we identified and postulate on the source of these gaps. The identification of these gaps and potential explanations provide the justification for drawing in outside literatures and our conclusions about how to advance the literature of agricultural health and safety on automating farms and a warming planet.

We identified three primary gaps in the current literature. The first limitation is that much of the research examines environmental change, technological change, and health impacts as separate, rather than as co-constituting, forces. This is a significant problematic, as other outside research makes the convincing case that environmental change, technological change, and health impacts are all tightly linked outcomes (see below sections on environmental social science in particular). For example, we might consider research on the production of new avenues of agriculture, and the working quality and decision-making capacities of agricultural communities. Such a consideration might show how suites of new technologies (sometimes glossed by researchers as Ag 4.0), climate change, and rural community dynamics together influence agricultural outcomes [85,86]. In other words, while our review was designed to detect the intersection of these forces, the majority of the studies we examined did not study these forces as directly related.

Secondly, few of these studies examine how these changes are distinctly socially embedded and therefore informed by social structures. Agricultural work is a distinctly social arrangement, and its contours, character, and outcomes are all informed not only by policy frameworks, but also by group attitudes, social norms, cultural expectations and socio-economic inequality. For example, there are well-known inequalities in income and income security which stem from citizenship, legal status, and access to services [80]. However, these were seldom operationalized with the exception of a handful of studies focusing on dairy workers [6,83] and farmworkers [47,48]. While these studies represent an exception, they exemplify how research into social inequality and socially embedded practices can reveal numerous agricultural health and safety problems from workplace conditions to health services delivery [5,46,53].

The third gap is the lack of research addressing broad trends or extrapolating lessons from particular industries into agriculture more broadly. Much of the research instead confines itself to the particular parameters of specific industries, drawing out the health and safety considerations for dairy production [5,6,83], for example. Studies for this industry are robust without deducing trends or implications across industries. The critical scholarship thus becomes less broadly accessible or critical, as the results are not already in direct conversation with other researchers working in cognate industries. With lower attention paid to the intersection of technology change, climate change, and worker health and safety, a gap swallows the opportunity for the field of agricultural health and safety.

Filling such a gap could be addressed with a simple enjoinder to ‘pay attention’ and increase scholarly activity on the overlaps. However, a simplistic research mandate for this or that industry or specific population could potentially limit this work, a counterproductive approach particularly because researchers need a wider theoretical toolkit to seriously identify and analyse these complex intersections. Put differently, a small research conversation on the topic itself produces a second challenge: a lack of theoretical diversity in the research discourse. Without a broader scientific conversation and diverse theoretical approaches, the scope of inquiry has a small theoretical and methodological toolkit for addressing the concerns. Scholars often translate approaches from other areas of inquiry without context or ‘start from scratch’. We suggest that, considering the way other disciplines, including science and technology studies (STS), rural sociology, and environmental social science, address subsections of these concerns, agricultural health and safety researchers must also look further afield to address the intersection of agricultural automation and climate change.

## 4. Social Science’s Contributions to the Environmental-Technological Change Intersection and Agricultural Worker Health

While social scientists have not traditionally played an important role in the field of agricultural health and safety, three disciplines have made important contributions at the intersection of environmental and technological change: rural sociology, STS, and environmental social science. We suggest that these contributions can be leveraged to deepen our understanding of agricultural worker health and safety as it pertains to climate change and automation and to provide resources for future research inquiry. We now describe these disciplines drawing from our own background. We recognize that each have their own research communities and agendas. As such, we do not wish to misrepresent our discussion as authoritative—but rather as a provocation to highlight the value some specific social science research communities hold regarding the problem of agricultural health and safety and the way these dynamics are theorized by the scientific community.

### 4.1. Contributions from Rural Sociology

Rural sociology arose in tandem with the land-grant university system and has long used a sociological lens to develop both original and applied research on rural society with an emphasis on agricultural change and natural resource use [87,88,89]. Rural sociology also has a long history of situating health, agriculture, technology change, and climate change, though rarely across all four of these domains [9,10,21,90,91,92,93,94]. One of the strengths of Rural sociology is the discipline’s willingness to adopt ‘toolkits’ from other disciplines based on the diverse and intersecting needs of this interdisciplinary field. One particularly relevant adoption includes adding an agricultural technology approach from the STS field [10,95,96], which we will look at more closely below. Rural Sociology also adopts mainline sociological approaches to understanding power and politics in rural food production [97,98] and public health approaches to rethinking rural health contexts [99]. While there are many frameworks that this body of scholarship may offer, we suggest two productive avenues for studying the intersections of automation, environmental change, and health in agricultural practices which we introduce and then further examine in the conclusion.

Rural sociology retains disciplinary roots in small communities embedded in and thus immediately dependent on the natural landscape, the fields has always had to draw empirical and theoretical connections between society and place [89,100]. Pragmatically, this has meant drawing relationships between place as a materiality, meaning its physicality has social ramifications—alongside the overarching or embedded social structures (which we typically think of as social, such as policy frameworks and class roles) that govern society in these contexts, be it community resilience, farmer wellbeing, agricultural sustainability, or rural development [37,53,93,101,102]. Rural social science is not siloed inside sociology alone; a body of anthropologists at the margins of agricultural health and safety research likewise have shown how place-based, ethnographic research into the specifics of agricultural communities and praxis help understand environmental and technological change on the farm and the health and wellbeing of farming communities [103,104,105,106,107,108]. Yates Doer in particular critically re-assesses how health research of rural sites imagine and inform social determinants and argues that the social nature of such health determinants also receive more consideration [108]. Such connections are much needed for future research, which would benefit from considering theoretical approaches that account for the interrelationship between place and materiality as rural sociology does, instead of considering as separate factors, or occasionally, as an addition *layer* that rests beneath or above material concerns.

### 4.2. Contributions from STS

STS has always been an interdisciplinary field, drawing on useful frameworks from other areas of inquiry, not unlike rural sociology. Central to this body of work however, are the methods and theories by which STS scholars have situated material environments, including the built and natural worlds, alongside societal outcomes ranging from health [109,110,111], to industry [112,113], to environmental outcomes [114,115,116]. Similar to rural sociology, which embeds society in the landscape, STS provides a useful way of relating the materiality of technology, in this case, agricultural technology and automation, with societal structure that inform their development and which are informed by their specific technical arrangement, a process called co-production [117,118,119,120]. For example, recent research in the development of robotics for the apple industry have shown how farmer’s considerations about robotics, and their anticipation of new norms around agricultural robotics, inform development of agricultural robotics and the adoption patterns that result—indicating how the technologies and societal norms around those technologies ‘make’ each other [121].

Recognizing that society engages the making of technological futures, and the reciprocal nature of this process, emphasizes how technology shapes societal outcomes. In agricultural work, we might consider how economic ideologies inform the ‘need’ for farms to scale, while at the same time, automated ‘smart’ technologies and data-driven practices such as precision agriculture enable and encourage that scaling, co-constituting one another [10,58,122,123,124]. These constructions are not imposed from above. Interdisciplinary approaches reveal that practitioners at all levels engage in this making of knowledge and therefore the resulting social, material, and lived outcomes. Mol et al., use such a framework in the particular case of nurses who ‘tinker’ with medical technology arrangements of hospital rooms to improve care outcomes, rethinking and re-making knowledge [111]. Likewise, when considering agricultural health, technology, and environmental change, an STS approach highlights not just the politics of knowledge production from the top, but also its interaction and re-interpretations with all those who interact with those technologies, such as the many farmworkers engaged with machines, plants, and animals on the farm [49,125].

Above, we noted that, in some US and European settings, STS concepts have already demonstrated useful synchronicity with rural sociological considerations and with environmental sociological approaches. In many ways, these demonstrations indicate the utility of these theories across fields and their potential use value to agricultural health and safety and other occupational scholarship [116,117,118,126,127]. Consider, for example, Lundstrom et al.’s work on care in dairy agriculture where automated milking systems are deployed [83]. This was one of the few studies our literature review identified as applying an STS approach to agricultural safety. Similar to many STSs and STS-inflected works in agriculture, the scholarship borrows, in this case, from social psychology’s Activity Theory to consider, in more typical STS fashion, the relationship between attitudes and beliefs of farmers and material changes on the farm. In this case, the researchers found that the deployment of milking systems had, for some farmers, improved overall wellbeing when said farmers could adapt to a continuous learning frame. Thus, their study demonstrates that automation in dairy and the changing ecological pressures on agriculture intersect and impact farm work.

### 4.3. Contributions from Environmental Social Science

Environmental social science, but particularly environmental sociology, has long connected environmental change, the uniquely material operations of societies in their physical environments, and the role of these societies in environmental change. While this research primarily discusses environmental sociology, the field of research on environmental justice is itself interdisciplinary, with practitioners from backgrounds in many disciplines but with a close relationship with political ecology that has an impact on anthropology and human geography [128,129]. From the beginning of this field, researchers have shown an interest in how social hierarchies such as those based on race, class, gender, ethnicity, and nationality produce inequality (e.g., polarized status, income), with particular interest in inequitable experiences with pollution and with a focus on urban settings [130,131,132]. Core areas overlap with farm labor, such as Arcury’s work, identified through our literature review [45]. Employing environmental justice (EJ) framings, Arcury examines the inequalities farmworkers face, including the politics involved in pesticide exposure [47]. This approach has been useful for understanding how environmentally embedded practices, such as agriculture, energy production, and city design, are impacted by social structures and particularly those that result in place-based inequalities [133,134,135,136]. In many ways, we might think of each of discipline discussed in these three subsections as clarifying how the intersection of automation, health, and climate change in agriculture is embedded in material domains, of place, technologies, and environments. The environmental social science scholarship offers two key considerations for use in agricultural health and safety scholarship.

The first is a theoretical framework for describing how societal norms of production and growth reproduce inequality in environmental outcomes. While multiple approaches exist to describing this phenomena, a popular approach in Environmental Sociology is the study of ‘treadmills’ by which production in industry creates stable reifications of inequalities with negative societal or environmental outcomes [137,138]. Somewhat different than the ‘treadmills’ discussed in rural sociological discourse, these approaches articulate the way neoliberal markets forms loops of production that reproduce environmental harms without developing off-ramps or environmentally beneficent improvements in the productive system. We suggest that this may be a useful theoretical ‘tool in the toolkit’ as it provides a way for relating, or differentiating, the extent to which automation and climate change exacerbate, reinforce, or ameliorate the treadmills of production existent in previous formations of agriculture over the last century—particularly as the treadmill approach is useful for examining the offloading of harms and the production of inequality in a wide variety of industries.

This leads to the second theoretical approach, closely paired to the first: the maintenance and production of environmental health outcomes resulting from unequal exposure to chemicals, pollutants, and other environmental harms—an approach often applying EJ theory [130,131,132,136,139,140]. In these approaches, the structures that (re)produce inequality are studied with the explicit motivation to ameliorate these outcomes. This renders EJ approaches specifically well-adapted to producing research with policy-level and intervention-level insights—a key outcome goal of many agricultural health and safety research practitioners. Unlike much agricultural health and safety research, which focuses on farm operators and owners as key collaborators and recipients of interventions, an integration of justice-oriented approaches such as EJ could provide frameworks for better involving and including disenfranchised and under-represented populations such as farm workers in research practices and in intervention outcomes.

## 5. Conclusions

Rapid automation coupled with the effects of climate change raise alarms for the safety and wellbeing of those who work in agriculture. Consistent with review frameworks, we took a systematic but exploratory approach to review existent literature and then drew connections with synchronous outside disciplines drawing on our teams’ expertise. We found that while the agricultural health and safety field has studied the roles of automation and weather in health and safety outcomes, we argue that rapid automation and climate change present novel challenges connected to unpredictability. For example, current research has adapted to consider the linear progression of higher heat, but the many dynamic outcomes that are difficult to predict as mechanized practices shift, new technologies are adopted, and less predictable weather events occur with greater frequency. Science based interventions in agricultural health and safety will require research that is adaptive, inventive, and future-oriented in order to observe and explain the dynamic risks associated with these novel pressures in a timely manner. In this article, we leveraged a review of 137 articles to understand how links between agriculture, technology, and climate change as relevant to safety and health have been studied. Overall, we identified three key themes in the literature: (1) the adoption of adaptation strategies including automation and climate-smart agriculture in response to climate change, (2) the discrete causes of farm injuries under climate change and technological change, and (3) a discussion of society, care, and wellbeing to the future of automated farm work with a primary focus on dairy farmers and farmworkers and the impact of automation in the industry.

While environmental and rural social science scholars have noted the social embedded dimensions of technology and environmental change on the health and safety of those who work in agriculture [16,17,41,42,45,80], we note a small number of studies examining these dimensions. Furthermore, in addition to being socially embedded, environmental change, technological change, and health impacts are co-constituting forces, a key contribution and recognition offered by STS researchers [141]. However, we note that the agricultural health and safety field still largely examine these forces as separate. To address these gaps, we suggest that rural sociology, STS, and Environmental Social Science provide relevant tools given their long-standing traditions examining the socially embedded practices of food producing communities alongside the co-constituting forces of environment, technology and society. Drawing from the disciplinary frames we brought into the conversation above, we suggest three avenues of further inquiry to make progress towards the research gaps in the agricultural health and safety literature that we identified.

First, we suggest that increased attention to the multiplicity of this experience as relevant to the generalized agricultural regime in qualitative research in rural places will be required to identify new determinants of worker health and wellbeing. This avenue for inquiry has broad relevance to, and could draw heavily from, the methods, theories and discourses being pushed forward in rural sociological research that examines the place-based examples of social structures in rural locales. For example, Legun and Burch’s work [121] examines the particular case of apple producer’s adaptations in anticipation of new robotics, yet the insights they provide our grounded in in broader environmental and agricultural theories, and their results characterize both a component of the agricultural tableau through a particular industry in a particular place, but they also demonstrate insights that have relevance beyond the discipline.

Second, continuing to develop empirically grounded, industry-specific research is key, relating themes, trends, and theories between industries will also be essential in the dynamic agricultural landscape of the 21st century. Furthermore, more robust research into the politics, outcomes, and social structures engaged in the making, deployment, and practice of new technologies on farm. This work will be key both for understanding the contemporary impacts of climate change and technological change but also to theorize and produce anticipatory scholarship on the future trajectory of farm work and its health and safety outcomes. Inquiries and research projects in this area have significant overlap with STS scholarship and the theories and approached introduced in our discussion have impacts for potential research in this area. See, for example, Mol et al.’s work on care and practice on farms and in healthcare settings [111].

Last, we suggest that increased attention on the shared inputs and impacts between society and environment in health outcomes on dynamically shifting farms will be key for understanding the longevity of our agricultural system, its (social) sustainability, and its role in (re)producing (in)equality. For example, consider Arcury and Holmes’ respective work on the social and health support (or lack thereof) or Klerkx et al.’s work on the social sustainability impacts of digital and automated agriculture [46,48,49,84,86,111]. Again, this has significance in environmental, sociological and EJ approaches, and onboarding these theoretical frames can be the basis for productive inquiry in agricultural health and safety.

In each case, we suggest that expanding the literature will benefit by borrowing from the valuable work being carried out in other fields to dynamically adapt our research to the quickly changing landscape of automating agriculture on a warming planet. In and out of itself, this is an integral research goal if we are to better understand and design programs, resources, and policies to promote safer and healthier farm work throughout the 21st century.

## Data Availability

Not applicable, data may be retrieved from the databases described in the review.
